# Bilateral stress fracture of the femoral neck in association with simultaneously developing osteonecrosis of the femoral head: a case report

**DOI:** 10.1186/s13256-021-03198-2

**Published:** 2021-12-22

**Authors:** Tomofumi Nishino, Hisashi Sugaya, Naoya Kikuchi, Yu Watanabe, Hajime Mishima, Masashi Yamazaki

**Affiliations:** Department of Orthopaedic Surgery, Faculty of Medicine, University of Tsukuba, 1-1-1 Tennodai, Tsukuba, Ibaraki 305-8575 Japan

**Keywords:** Femoral neck stress fracture, Osteonecrosis of the femoral head, Osteoporosis

## Abstract

**Background:**

Femoral neck stress fractures are rare and often recognized as overuse injuries that occur in young athletes or military personnel. A case following osteonecrosis of the femoral head is quite rare; even more uncommon is its occurrence in the bilateral hips. Magnetic resonance imaging has been established as the preferred tool for diagnosing nondisplaced femoral neck stress fracture due to overuse injury. Magnetic resonance imaging was also useful to detect the initial lesion even in this case, although the etiology was different between overuse injury and insufficiency fracture.

**Case presentation:**

A 41-year-old Japanese woman diagnosed with bilateral early stage idiopathic osteonecrosis of the femoral head was observed non-weight-bearing as much as possible using a stick. However, her pain and difficulty in walking progressed. Bilateral femoral neck stress fractures were subsequently detected by magnetic resonance imaging. The fracture initially appeared as a spot of bone marrow edema at the medial site of the femoral neck, and then developed into a fracture line. The patient underwent internal fixation of both hips with sliding hip screws to stabilize the stress fractures. In addition, the preparatory reaming served as core decompression of the femoral heads, as well as being treatment for osteonecrosis. Her bone mineral density and 25-hydroxy vitamin D values were low for her age. We administered eldecalcitol and teriparatide acetate. Her symptoms mostly improved, and the fracture lines and necrotic lesions on magnetic resonance imaging reduced at 5 months after the surgery.

**Conclusions:**

Bilateral femoral neck stress fractures are a very rare condition and are often missed. It is important to listen to the patient’s complaints and perform an appropriate examination. We encountered a case of bilateral femoral neck stress fracture that occurred in a patient with early stage osteonecrosis of the femoral head, and were able to observe progression of stress fracture since before fracture occurred. This is considered to be the first report to capture imaging changes before and after the onset.

## Introduction

Femoral neck stress fractures are an unusual condition. The occurrence of bilateral femoral neck stress fracture in a patient with bilateral osteonecrosis of the femoral head is even more uncommon [1–6]. Stress fractures are the result of repetitive mechanical stress on the bone and may occur in normal and abnormal bones. Causes of abnormal bones include pathological fractures due to hormonal and metabolic abnormalities. Although stress fractures associated with overuse injury have been reported [7–9], there are few reports on the etiology and mechanism of the simultaneous occurrence of stress fracture and osteonecrosis of the femoral head. We report a case of progression of stress fracture with early stage osteonecrosis of the femoral head, including diagnosis with magnetic resonance imaging (MRI).

## Case presentation

A 41-year-old Japanese woman presented to our clinic with a 4 month history of bilateral groin pain and right buttock pain. Her right hip was more painful than her left hip. There was no history of trauma, alcohol abuse, or steroid use. Her medical history included iron-deficiency anemia diagnosed 2 years earlier, after which she had been on iron supplements. She had no fracture episodes, including fragility fractures.

Her height, body weight, and body mass index were 155 cm, 42 kg, and 18.7 kg/m^2^, respectively. She was able to walk for approximately 10 minutes without a stick, albeit at a slow speed. Limitations in the passive motion of her bilateral hip joint were observed thus: flexion, 100°, internal rotation 5°, external rotation 15°, and abduction 20°, on both sides. She was able to perform a straight-leg raise of the right limb with substantial pain. The neurovascular status of both lower extremities was intact. The Japanese Orthopaedic Association scoring system for the evaluation of hip-joint function (JOA hip score) was 46 points for her right hip and 56 points for her left hip. The score was based on a total of 100 points, comprising 40 for pain, 20 for range of motion, 20 for the ability to walk, and 20 for activities of daily living [10].

**Fig. 1:**
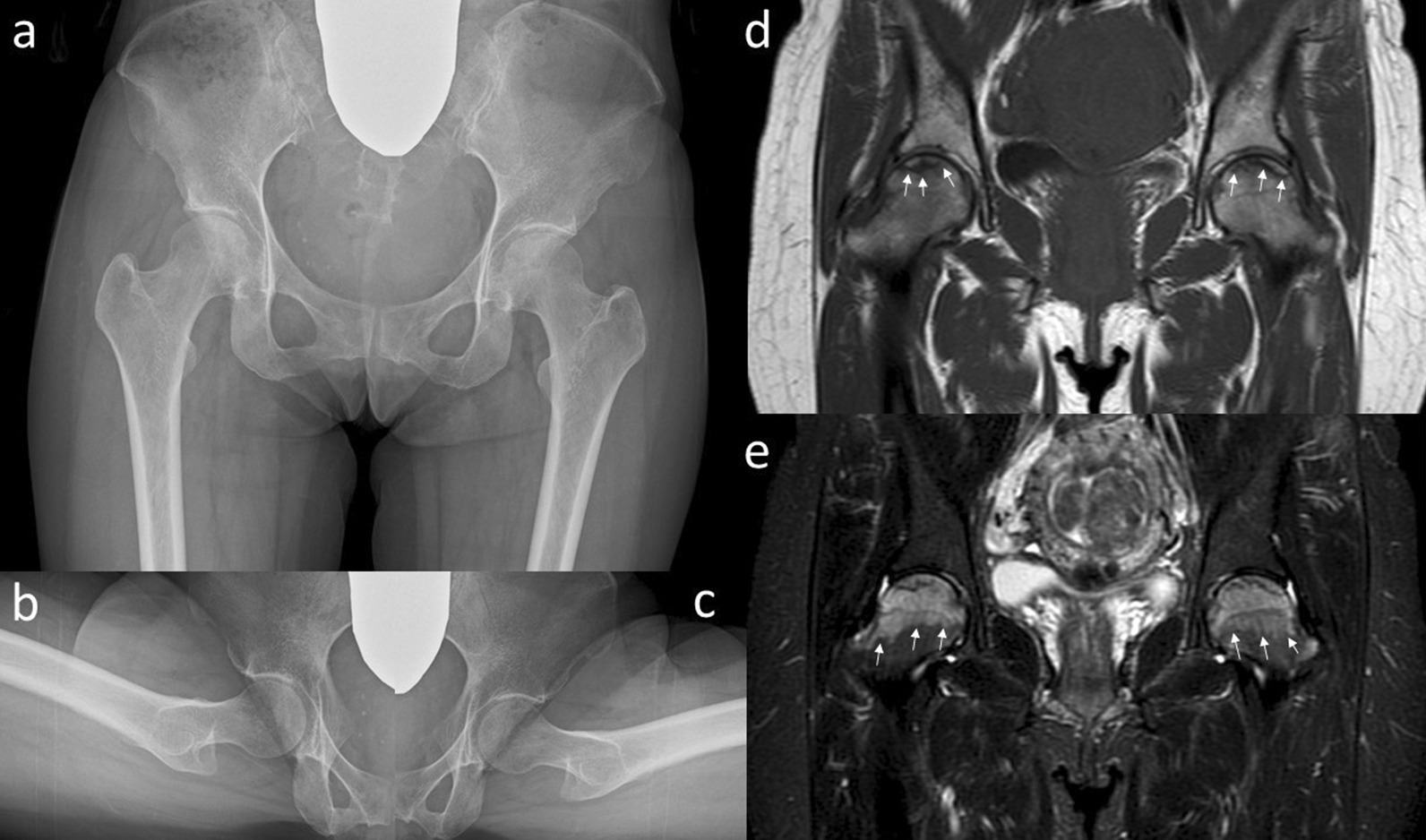
Plain radiographs **a**–**c** and magnetic resonance imaging **d**, **e** of the hip joint at the first visit. Anteroposterior **a** and lateral (Sugioka) (**b** right hip joint; **c** left hip joint) views demonstrate unremarkable findings. The coronal view of a T1-weighted image of the magnetic resonance imaging **d** shows the presence of a low signal line in the subchondral region of the femoral head (white arrows). A high signal region spreads to the femoral neck (white arrows) in the coronal view of a short tau inversion recovery (STIR) image of the magnetic resonance imaging (**e**)

**Fig. 2:**
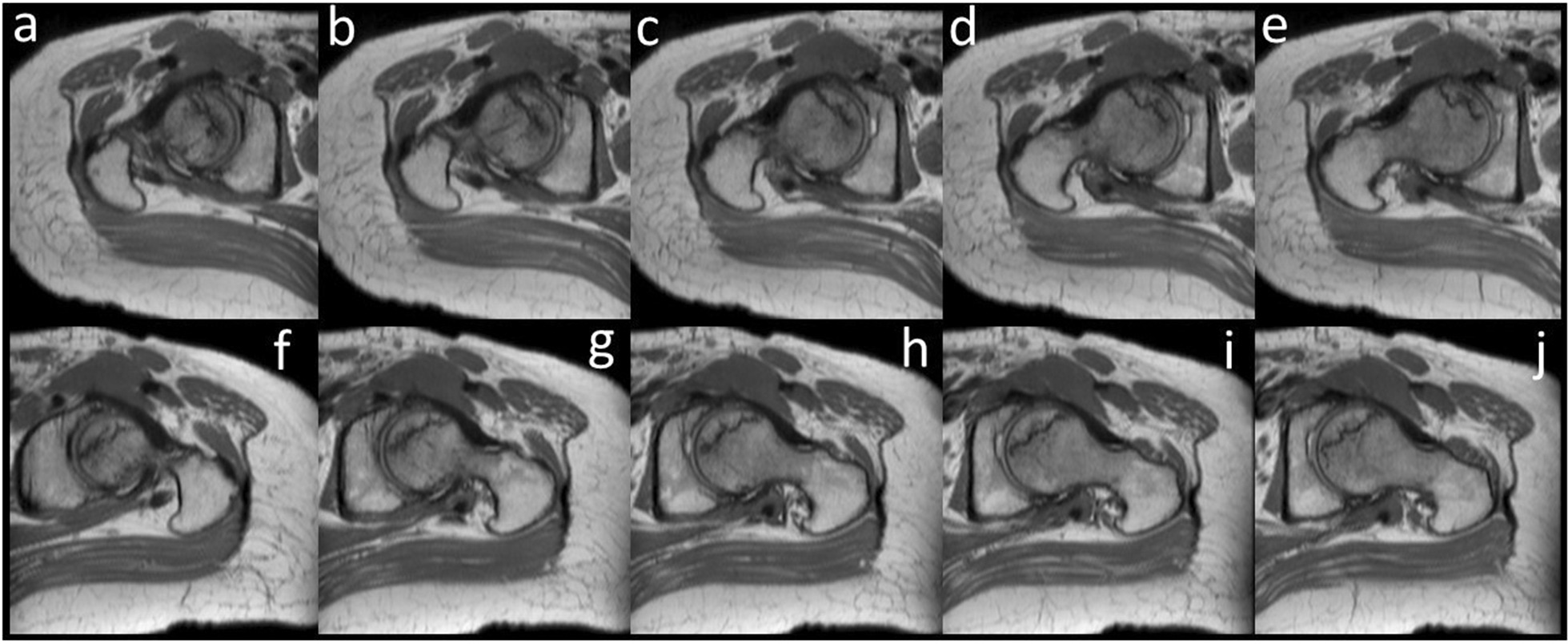
The oblique axial views of proton density weighted image. **a**–**e** are on the right side; **f**–**j** are on the left side; and **a** and **f** are on the most cranial side.

Standard radiographs of both hips (Fig. 1a–c) demonstrated no characteristic findings such as the crescent sign, sclerotic band pattern, and collapse of the femoral head, and no joint space narrowing was seen in either femoral head. MRI of both hips (Fig. 1d, e) presented a low signal line in the subchondral region of the femoral head in the T1 weighted image and high signal region in almost all of the femoral head in the short tau inversion recovery (STIR). The oblique axial views of the proton density-weighted image showed a low-signal sinuous line in the anteromedial region of the femoral head (Fig. 2). Dual-energy X-ray absorptiometry (DEXA) values were low in both femoral necks. Bone mineral density was 0.909 g/cm^2^ (T-score –0.9, Z-score −0.8) in the lumbar spine, 0.594 g/cm^2^ (T-score −1.8, Z-score −1.4) in the right femoral neck, and 0.529 g/cm^2^ (T-score −2.4, Z-score −2.0) in the left femoral neck. Laboratory findings were as follows: C-reactive protein (CRP) 0.03 mg/dl (normal range: 0–0.5 mg/dl); alkaline phosphatase 608 IU/l (40–150 IU/l); calcium 9.0 mg/dl (8.4–10.2 mg/dl); albumin 4.4 g/d (3.9–4.9 g/d); and hemoglobin 9.7 g/dl (12–16 g/dl). Bone turnover markers were as follows: tartrate-resistant acid phosphatase 5b (TRACP-5b) 463 mU/dl (premenopausal normal range: 120–420 mU/dl) and total procollagen type 1 N-terminal propeptide (Total P1NP) 72.7 ng/ml (26.4–98.2 ng/ml). We diagnosed bilateral osteonecrosis of the femoral head and classified it as stage 1 in both femoral heads, according to the Association Research Circulation Osseous (ARCO) classification [11].

**Fig. 3:**
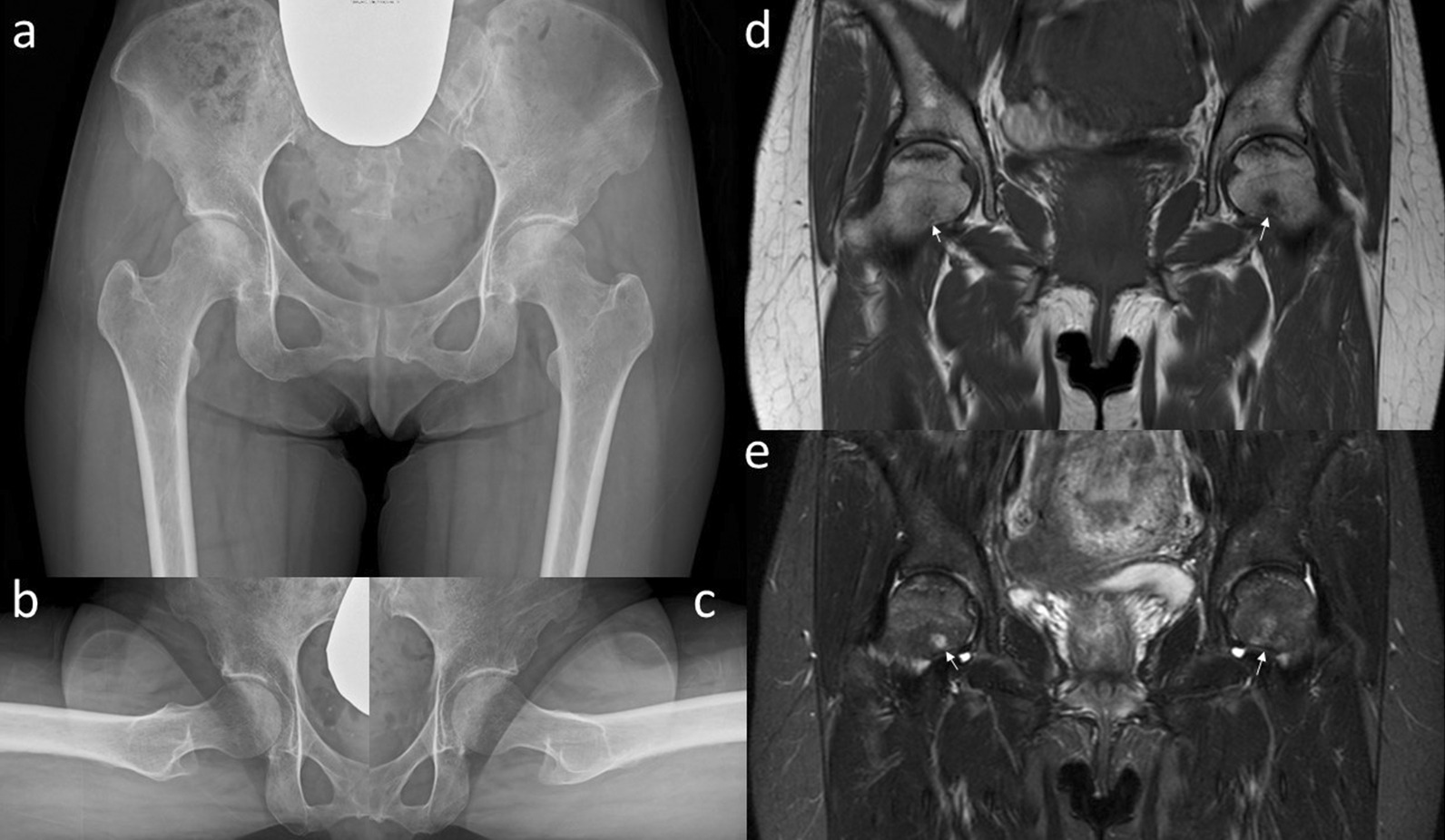
Plain radiographs **a**–**c** and magnetic resonance imaging **d**, **e** of the hip joint at 6 months after the first visit. Anteroposterior **a** and lateral (Sugioka) (**b** right hip joint; **c** left hip joint) views demonstrate unremarkable findings. The coronal view of a T1-weighted image of the magnetic resonance imaging **d** shows the presence of a low signal area in the subcapital region of the femoral head (white arrows). A high signal area appears in the subcapital region (white arrows) in the coronal view of an STIR image of the magnetic resonance imaging (**e**)

**Fig. 4:**
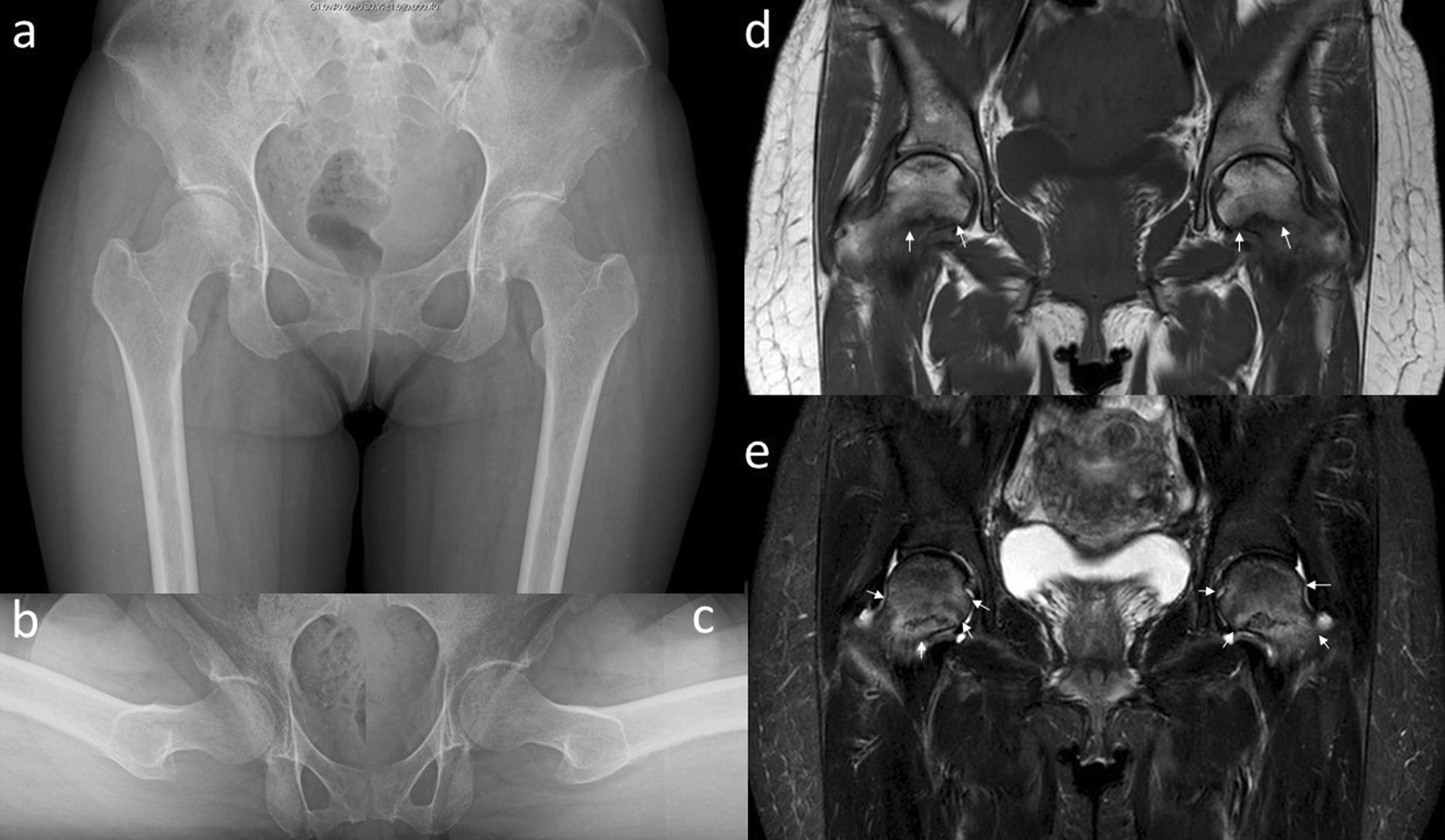
Plain radiographs **a**–**c** and magnetic resonance imaging **d**, **e** of the hip joint at 10 months after the first visit. Anteroposterior **a** and lateral (Sugioka) (**b** right hip joint; **c** left hip joint) views demonstrate unremarkable findings. The coronal view of a T1-weighted image of the magnetic resonance imaging **d** shows the presence of a low signal line in the subcapital region of the femoral head (white arrows). A high signal area appears around a low signal line (white arrows) in the coronal view of an STIR image of the magnetic resonance imaging (**e**)

**Fig. 5:**
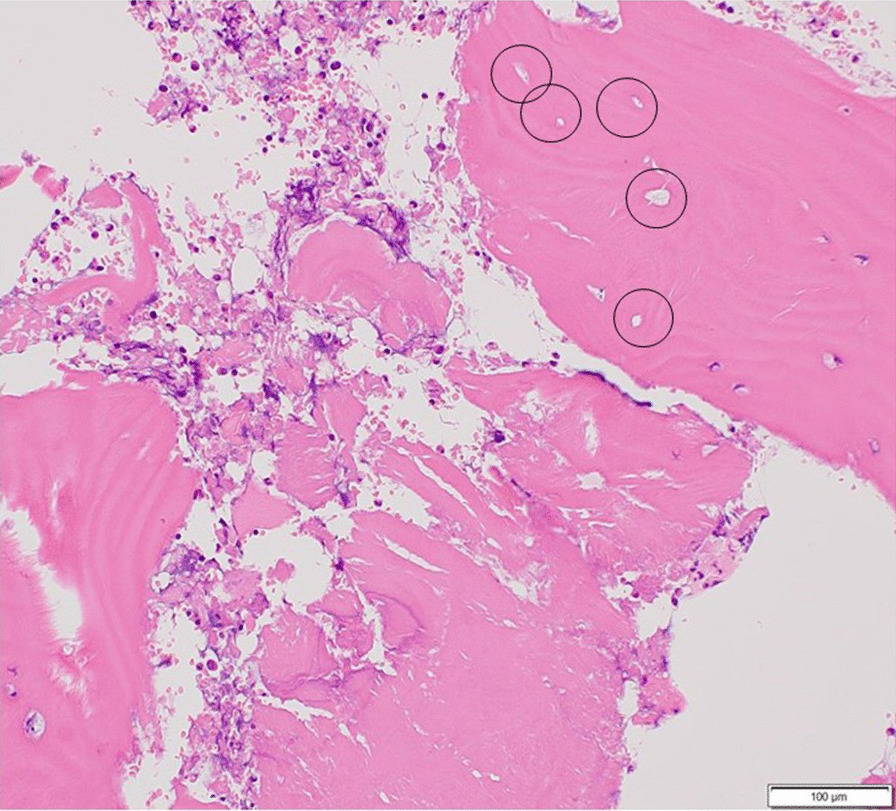
Photographs of the specimens obtained from reaming with hematoxylin and eosin stain. A histopathologic image of the necrotic region shows bone trabeculae with empty lacunae enclosed by a circle

**Fig. 6:**
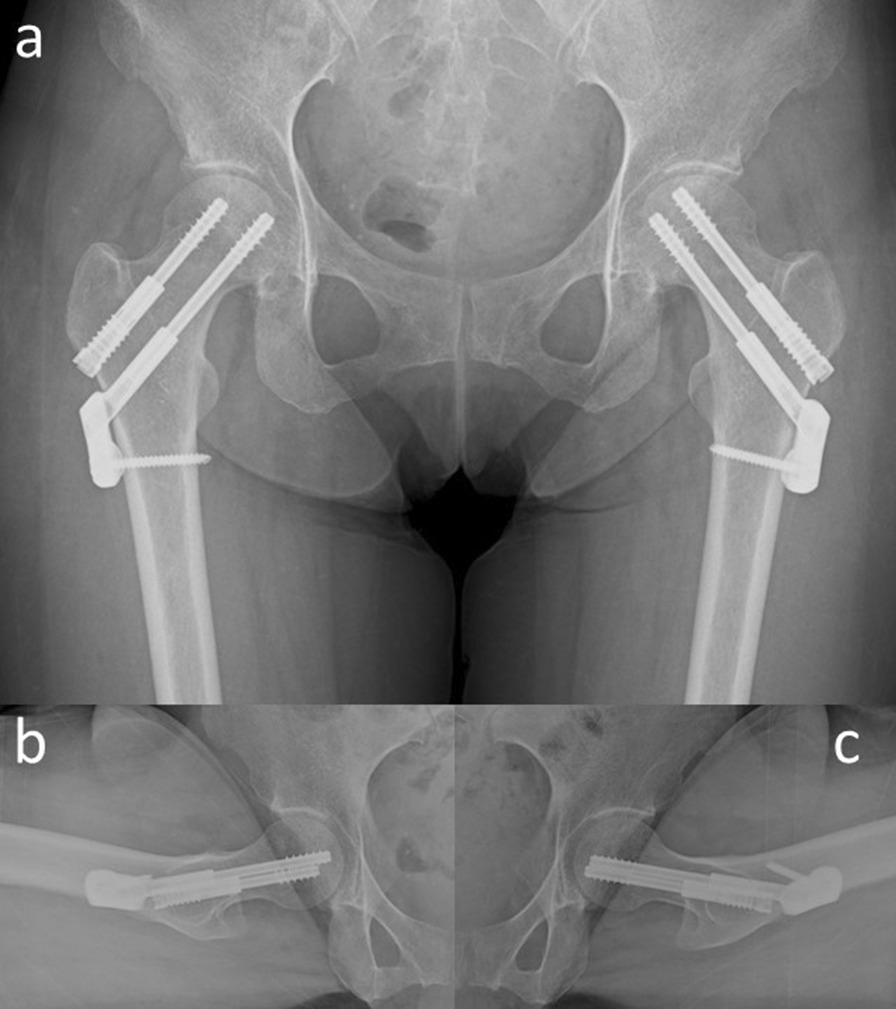
Plain radiographs **a**–**c** immediately after the surgery. Anteroposterior **a** and lateral (Sugioka) (**b** right hip joint; **c** left hip joint) views demonstrate the inserted sliding screws without fracture displacement

**Fig. 7:**
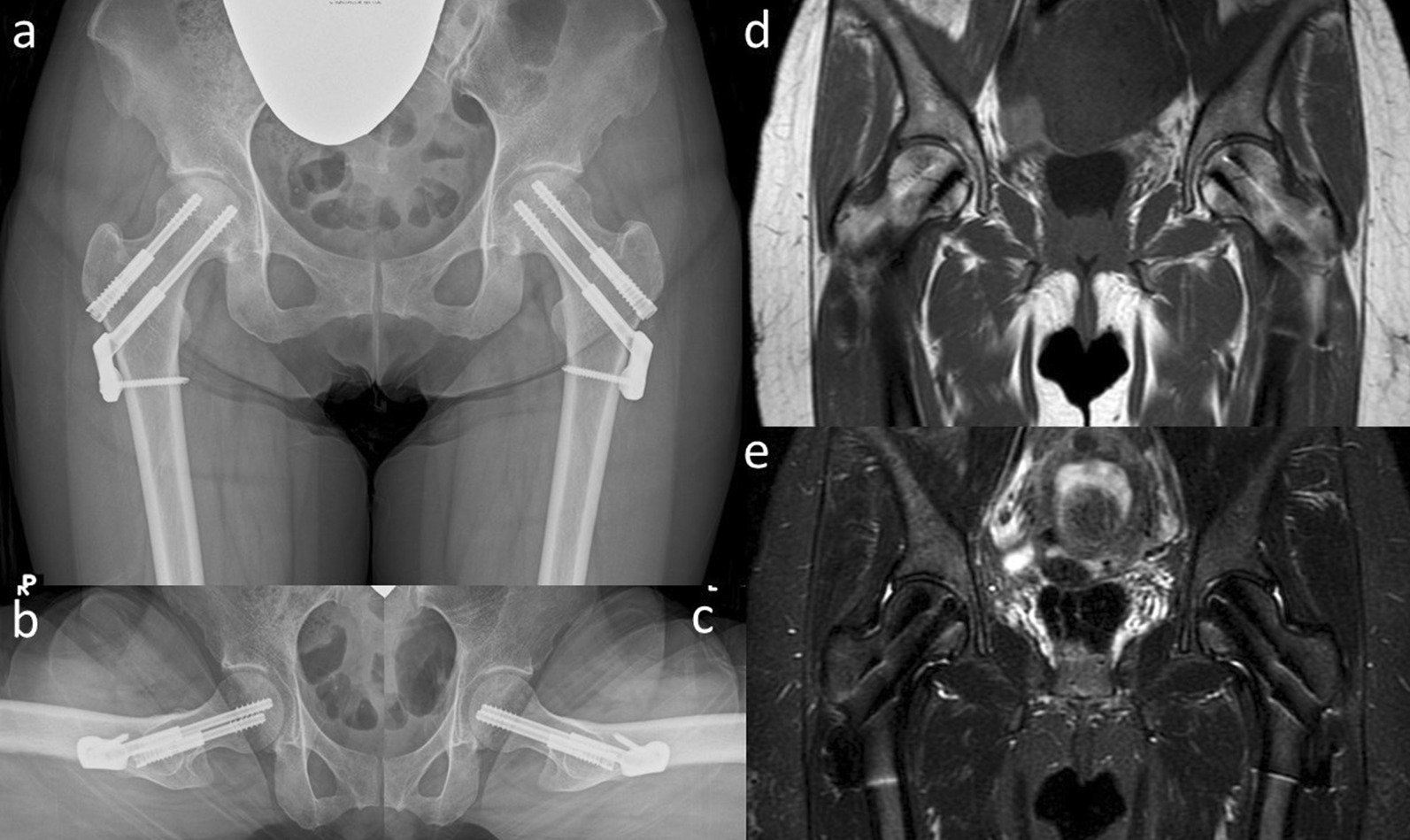
Plain radiographs **a**–**c** and magnetic resonance imaging **d**, **e** of the hip joint at 5 months after the surgery. Anteroposterior **a** and lateral (Sugioka) (**b** right hip joint; **c** left hip joint) views demonstrate no remarkable change. Although an artifact of the implant is seen in this image, the low signal line in the femoral neck still remains but is not enlarged in the coronal view of a T1-weighted image of the magnetic resonance imaging (**d**). Likewise, a high signal area disappeared around the femoral neck in the coronal view of an STIR image of the magnetic resonance imaging (**e**)

**Fig. 8:**
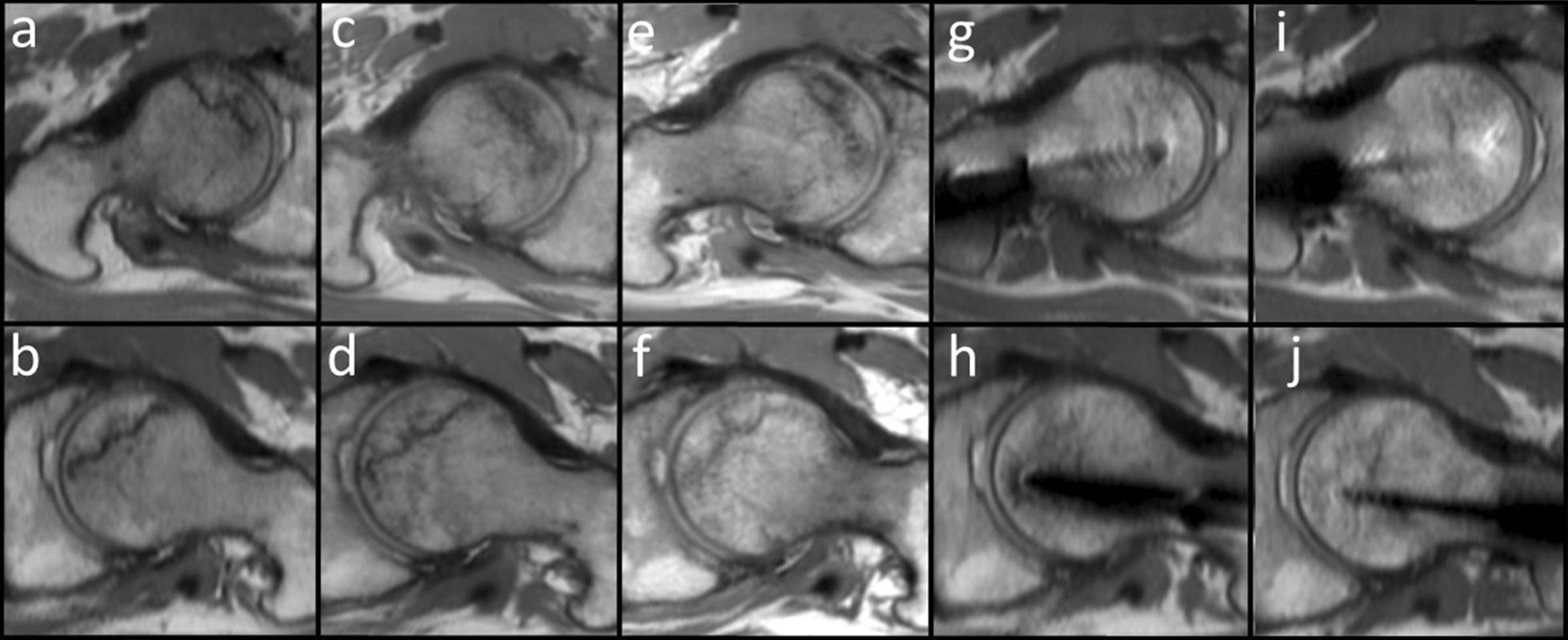
Oblique axial views of the proton density weighted images. **a** right femoral head at the first visit; **b** left femoral head at the first visit; **c** left femoral head 6 months after the first visit right; **d** left femoral head 6 months after the first visit; **e** right femoral head 10 months after the first visit; **f** left femoral head 10 months after the first visit; **g** right femoral head 2 months after the surgery; **h** left femoral head 2 months after the surgery; **i** right femoral head 5 months after the surgery; and **j** left femoral head 5 months after the surgery. Progress of the femoral head is shown sequentially. Although there is an artifact of the implant, the low signal area in the femoral head does not enlarge

The patient was instructed not to take as much weight as possible using sticks on the right side. Her symptoms improved immediately. However, 6 months later, her symptoms increased slightly without any traumatic episodes. Radiographic findings indicated almost no change (Fig. 3a–c). However, a slight signal change in the medial subcapital region was observed in the MRI scan (Fig. 3d, e). Her pain gradually increased thereafter, and 10 months after her first visit, walking became difficult. The JOA hip score decreased to 34 points in both hips; still, no changes could be observed in the radiograph (Fig. 4a–c). MRI of both hips showed a nondisplaced subcapital fracture on the medial side of both femoral necks, with bone marrow edema around the fracture (Fig. 4d, e). Because the cause of the fractures was not identified, the DEXA and bone turnover markers were measured again, and 25-hydroxy (OH) vitamin D was measured for the first time. All DEXA values decreased. The bone mineral density was 0.849 g/cm^2^ (T-score −1.5, Z-score −1.2) in the lumbar spine, 0.527 g/ cm^2^ (T-score −2.4, Z-score −2.1) in the right femoral neck, and 0.490 g/cm^2^ (T-score −2.7, Z-score −2.4) in the left femoral neck. TRACP-5b level increased to 607 mU/dl, and total P1NP decreased to 52.7 ng/ml. Her 25(OH) vitamin D level was 11.1 ng/dL and she was diagnosed with vitamin D deficiency. Based on the above results, our diagnosis was bilateral stress fracture of the femoral neck secondary to osteonecrosis of the femoral head. The patient underwent internal fixation of both hips with sliding hip screws (Dual SC screw system; Kisco, Kobe, Japan) to stabilize the stress fractures. In addition, the reaming performed before inserting of the sliding hip screw served as core decompression for the femoral heads [12, 13]. The specimens obtained from the reaming were examined histologically. Definitive findings of osteonecrosis such as bone marrow necrosis and loss of osteocyte nuclei in the femoral heads were observed (Fig. 5). Postoperative radiographs showed no evidence of displacement of the fractures (Fig. 6). We administered eldecalcitol 0.75 μg per day orally for vitamin D deficiency, and daily subcutaneous injections of teriparatide acetate. In the immediate postoperative period, the patient began to bear weight as tolerated with the use of an assistive device bilaterally. One month postoperatively, she was able to walk without pain and used a cane part time. She eventually regained full walking ability without a cane 3 months after surgery. Furthermore, her JOA hip score improved to 90 points in both hip joints at 5 months after surgery. Radiographs showed no evidence of recurrent stress fracture in the femoral neck or progression of osteonecrosis (Fig. 7). Sequential oblique axial MRI showed that the necrotic region of the femoral head had decreased 5 months after surgery (Fig. 8).

## Discussion

Several cases of bilateral stress fracture of the femoral neck following osteonecrosis of the femoral head have been reported [1–6]. We encountered a case of bilateral femoral neck stress fracture that occurred in a patient with early stage osteonecrosis of the femoral head, and were able to observe progression of stress fracture since before fracture occurred.

Our patient had no risk factors for osteonecrosis, including history of alcohol abuse or steroid use. Idiopathic osteonecrosis of the femoral head was most considered because a middle-aged woman who has anemia was involved. She was observed non-weight-bearing as much as possible because of the involvement of the bilateral hips at the early stage. However, her pain increased, and femoral neck stress fracture was detected by MRI during progression. The fracture appeared as a bone marrow edema at the medial site of the femoral neck, and subsequently progressed to stress fracture. It was classified under the compression category according to the Devas classification of femoral neck stress fracture [14]. Conservative treatment was provided at first, but the pain and disability steadily progressed. Finally, internal fixation was performed.

Stress fractures are the result of repetitive mechanical stress on the bone, and may occur in normal and abnormal bones. In terms of bone quality, Pentecost *et al*. classified stress fractures into three groups: fatigue fractures, which appear in bones with normal strength; insufficiency fractures, which appear in fragile osteoporotic bones; and pathologic fractures, which appear in fragile bones following tumor invasion [15]. In this case, her low bone mineral density and vitamin D deficiency might be associated with this etiology. We believe that vitamin D deficiency is a risk factor for insufficiency fracture and may lead to nontraumatic femoral neck fracture in nonelderly patients who have no other underlying risk factors for bone fragility [16]. Therefore, this was considered an insufficiency fracture using Pentecost’s classification.

When the region of osteonecrosis is large, it can easily lead to stress fracture in the femoral neck: the underlying interface of necrotic and viable bone where sheer stress is applied [1–4]. However, the early stage of osteonecrosis of the head passes adiabatically, and is often difficult to diagnose by radiograph only; therefore, some cases are diagnosed only after the occurrence of stress fracture [5, 6].

In our patient, the necrotic region was small and localized in the subchondral region, classified as stage 1 in both femoral heads; however, bone marrow edema involved almost the entire femoral head. Therefore, transient osteoporosis of the femoral head and a subchondral insufficiency fracture of the femoral head were considered as differential diagnoses. However, osteonecrosis of the femoral head was diagnosed based on the pathology of the specimen harvested during the operation. Because she had severe symptoms around the hip joint for large marrow edema, follow-up was possible from an early period using MRI.

MRI was established by Shin *et al*. as the imaging modality of choice for the diagnosis of incomplete femoral neck stress fracture [7]. Several key features were established in the initial MRI that can guide treatment and prognosis: osseous edema, the presence of a fracture line, and hip effusion. There is a subset of patients with focal edema without a fracture line, which is commonly referred to as a stress reaction [8, 9]. In this case, the stress fractures appeared as bone marrow edema through the interface between edema and viable bone at the femoral head–neck junction as an initial finding. Even if there was a difference in etiology between fatigue fractures in young athletes and insufficiency fractures in fragile patients, the MRI findings were similar to each other.

Surgical treatment is not usually indicated for a unilateral medial stress fracture of the femoral neck because the compression type according to the Devas classification is considered stable. Treatment generally consists of limited weight bearing until the fracture heals [9, 17–19]. However, given the bilaterality of the fracture, the patient’s activities of daily life became difficult. The pain gradually increased as the fracture progressed, and her daily activities deteriorated. The fracture line at the compression site exceeded the center of the neck in the MRI [9]. In addition, the history of osteonecrosis suggested that open reduction and internal fixation was preferable to nonoperative treatment. Although there are few established recommendations for treatment for such a rare case, Zuckerman* et al.* recommended internal fixation to provide not only pain relief but also the possibility of immediate walking with weight bearing for a similar case [6]. This was achieved early after surgery in our case, and the patient was satisfied.

In addition to stress fractures, the accompanying osteonecrosis of the femoral head in this case was another problem. Nevertheless, by treating the patient with sliding hip screws, we were in effect performing core decompression of the both hips, which might have provided effective treatment of the osteonecrosis while stabilizing the fractures. From the results of this alone or with similar cases, the necrotic region seems to reduce over time after surgery.

One of the limitations of our study is the lack of endocrine search for the cause of osteoporosis. We did not perform thyroid and parathyroid tests in our institute. In addition, osteoporosis treatment should have been started when the decrease in bone density and insufficiency fracture were found, but it was delayed due to the patient’s refusal.

## Conclusions

We encountered an extremely rare case of bilateral femoral neck stress fracture that occurred in a patient with early stage osteonecrosis of the femoral head, and were able to observe progression of stress fracture since before fracture occurred.

## Data Availability

Not applicable.
